# The Cancer Experience Map: An Approach to Including the Patient Voice in Supportive Care Solutions

**DOI:** 10.2196/jmir.3652

**Published:** 2015-05-28

**Authors:** Leslie Kelly Hall, Breanne F Kunz, Elizabeth V Davis, Rose I Dawson, Ryan S Powers

**Affiliations:** ^1^HealthwiseBoise, IDUnited States

**Keywords:** mHealth, patient participation, neoplasm, public policy, Internet intervention, cancer survivors, online interventions

## Abstract

The perspective of the patient, also called the “patient voice”, is an essential element in materials created for cancer supportive care. Identifying that voice, however, can be a challenge for researchers and developers. A multidisciplinary team at a health information company tasked with addressing this issue created a representational model they call the “cancer experience map”. This map, designed as a tool for content developers, offers a window into the complex perspectives inside the cancer experience. Informed by actual patient quotes, the map shows common overall themes for cancer patients, concerns at key treatment points, strategies for patient engagement, and targeted behavioral goals. In this article, the team members share the process by which they created the map as well as its first use as a resource for cancer support videos. The article also addresses the broader policy implications of including the patient voice in supportive cancer content, particularly with regard to mHealth apps.

## Introduction

### Background

“Patient voice” is a term that has become more common in health care contexts. It is often used to describe a compilation of many patients’ expressed feelings, concerns, and experiences during an illness. While “patient voice” sounds singular—as if all of these voices had somehow reached a consensus and blended into one voice—it is plural in that this “voice” reflects a span as wide as the vastness of human experience and response. For the purpose of this article, “patient voice” is defined as the perspective of a cancer patient, and acknowledging the voice of the cancer patient is synonymous with having a deep understanding of how cancer affects a person as a human being, including in physical, emotional, psychosocial, and spiritual ways.

When our team, consisting of a medical writer, an oncology content specialist, and two user experience researchers, set out to concretely represent the voice of the cancer patient, our goal was to build a tool for our company’s content developers. We call this new patient-voice model a “cancer experience map”. We believe that, in this time when technology makes very little seem impossible, the usefulness and long-term, proven success of mobile health apps (mHealth apps) will depend on the app developers’ willingness and ability to tailor their apps to the cancer patient. Here, we offer the cancer experience map as a new tool for mHealth app developers.

### Mobile Health Apps: Benefits and Barriers

Mobile health apps (mHealth apps) not only can be a source of support for people who have cancer but also can improve their quality of life. During cancer treatment, this support can help a cancer patient manage health-related issues and promote better patient-provider communication, collaboration, and shared decision making [1]. After cancer treatment, an mHealth app can ease this transition by helping him or her deal with lingering side effects, such as insomnia, or make health behavior changes, such as trying a new exercise routine [2]. Also, mHealth apps that are online coaching programs can benefit family or friends who are caring for a person with cancer—lightening their burden, increasing their coping skills, and improving their mood [3].

Amidst all this promise, there are some real-world limitations. A systematic review of mobile phone apps found that while there are hundreds of apps covering a range of cancer-related support, there is a lack of evidence on how they are used and how effective and safe they are [4]. There is no index or repository where a person can go to see what apps are available, nor is there a standard rating system that includes reviews by cancer patients who have tried these apps. In addition, mHealth apps in the United States are subject to legal implications, so health care providers and app developers need guidance to comply with regulations [5].

Many barriers have been identified. A recent review of mobile phone apps for breast cancer noted as a barrier the lack of consumer confidence in these apps and pointed out the need for a robust framework for identifying high-quality cancer supportive care apps that could be used by patients and their medical providers [6]. The technology limitations of mobile devices pose another barrier, and app developers are advised to use cancer patients to test their apps and then implement the design and functionality features recommended by these patients [1]. The unmet supportive care needs of cancer survivors after treatment are also a barrier that is becoming increasingly more evident [7].

### Capturing the Cancer Patient Voice

How can researchers and others who are interested in building mHealth apps for cancer supportive care know what cancer patients need the most? It may seem obvious that the first step would be to ask cancer patients what they need and want. But, surprisingly, the voice of the cancer patient is often absent from the conversations that lead to the design of supportive-care programs for this very population.

Some developers have invited cancer patients into their app development process, such as the researcher who created a real-time tracking tool for breast cancer patients and had patients try it out [8]. Others have created apps and then had cancer patients try them out, such as the researcher who made revisions after getting feedback from the adolescent cancer patients who tried out her game-type app for managing pain [9].

While the actual use and testing of mHealth apps with cancer patients during development and afterwards is essential, what about the initial steps in developing an app? A key starting point is a “rigorous evaluation of the consumer’s needs” [10]. Understanding “the nature and magnitude of the impact of cancer” is essential in planning supportive care [7].

Looking to gain entry into the mindset of cancer patients, our small team found ourselves at this very point when assigned the task of creating a suite of cancer support videos. In this paper, we share how we developed a representational model to reflect the cancer patient voice and how we used this model in our product development process. We also briefly discuss the broader concerns of the patient voice and recent steps that have been taken to represent that voice in health care public policy in the United States. The aim of this paper is to offer our process and the resulting cancer experience map as a resource for designers of mHealth apps specifically for this population. Our deepest hope is that ultimately cancer patients will benefit from the sharing of this story.

## The Oncology Content Specialist: Hearing the Patient Voice

When our health information company decided to create supportive cancer content, an early assignment came to the oncology content specialist and the user experience researchers. Our task was to create a representational model to support script development for cancer support videos and for future cancer content development.

As the oncology content specialist, I was responsible for providing information, resources, and insight about cancer and cancer patients to the team. For over 6 years, I had followed the medical news on cancer and had been tracking the “consumer experience” of cancer patients online in blogs and forums as well as in other media. While my work often involved reviewing oncology content for medical accuracy with an eye to the patient perspective, my initial meeting with the user experience researchers was the first time I was part of a project where the scope of the discussion was the entire range of the cancer experience. Two questions were paramount in the conversation: Who is the person with cancer? And what does a person with cancer experience?

I remember the day that they diagnosed me. I left the hospital, and I couldn’t find my car. It was in a parking garage. I literally was bumping into cars. I was so broken up. I couldn’t see where I was walking. It was just like, ‘Oh, my God. This can’t be happening to me’. [11]

Emotionally, you don’t drop to the bottom; you get thrown to the bottom. [11]

When asked to summarize my informal observations, I talked about the patient interview I had recently read in which a man described losing his car in the hospital parking lot shortly after finding out he had cancer. This story brought up the striking contrast between reading research *about* people who had cancer and reading what was written *by* cancer patients about their experiences. It had to do with hearing the voice of the cancer patient. Having cancer isn’t like having another kind of illness but is an experience that only those who have had cancer seem to truly understand. There is also a considerable gap in perspective that appears to exist between people who have cancer and those who don’t. Sometimes it seems as if people with cancer are members, albeit unwilling members, of an exclusive club, and those outside—those who haven’t been “thrown to the bottom”—are left wondering what is happening.

It is hard to describe how unsafe, angry, depressed, and betrayed by my own body I felt when first diagnosed with cancer. [12]

It was also fairly apparent from reading the stories of cancer patients that people don’t have uniform or parallel responses to having cancer. Individuals at one end of the spectrum experience empowering personality changes, while at the other end there are fiercely private individuals who are determined not to say anything about their cancer, who hope no one will find out.

A few weeks before my meeting with the user experience researchers, the medical writer [RSP] and I had talked about our hope that the cancer support videos would feature patients relating their experiences rather than actors reading a script. While our company’s process was underway for creating the necessary legal paperwork, we soon learned that the permission forms wouldn’t be ready in time for our project. With this change in plans, we now needed a representational model more than ever.

I was trying to find information about what treatments are available and things like that, but I kept on finding that every person is different. [13]

I can’t be the only person who fought cancer and will never say ‘Well, in the end, it was a gift’. [14]

## The User Experience Researchers: Building the Cancer Experience Map

### Overview

From the start of developing our cancer support videos, we planned to work with the oncology content specialist to develop personas to represent our target users. A persona is an archetype that is based on user research and that communicates user requirements [15]. Using a persona during product design helps writers and developers understand the information needs as well as the goals, challenges, motivations, hopes, and fears of their target audience.

### Researching the Persona

While personas are very common in product development, most companies treat them as proprietary internal collateral and don’t share them outside the company. We suspect that many cancer personas exist, but we located only a few limited cancer personas online. It seemed clear that approaching something as complex as cancer would need a wider view than the view that our established persona process provided. A cancer patient’s needs and experience change dramatically over the course of time. We felt that our traditional single-point-in-time persona format would not be enough. A point-in-time persona, even brilliantly rendered, could not adequately represent the cancer patient’s experience.

To gain insight into an approach that would inform our team and fit both immediate and longer-term needs, we engaged friends and coworkers to chart their perceptions of *their* personal cancer journeys. These exercises reinforced the complexity of the cancer journey and hinted at common points of experience. We decided that the similarities in the experiences of different kinds of cancer warranted our pursuing a universal cancer experience map.

The hardest part was the waiting. I wasn't sure what it was going to do—if it was going to rapidly expand or slow down . . . If you’re not sure what's going on in your body, it's hard to sit down and think about anything that's going on around you. [16]

After I got my cancer-free diagnosis, that's when I got depressed . . . Disease-free is the moment—it doesn't mean you're going to stay that way. Everyone around me was celebrating; they were happy, life was good, and I became completely depressed. [17]

We noticed that the timeline created from the discovered common points followed a clinical path. This wasn’t surprising, given that the experience of cancer is often closely tied to what is happening clinically—for example, getting a diagnosis or making treatment decisions.

With the stages in the timeline identified, we again teamed up with the oncology content specialist to collaborate on how we could keep the patient voice in the forefront. In addition to talking to cancer patients, we had found that actual patient quotes seemed to be our best option for accurately capturing the patient voice, and our preference was to get this information first-hand, rather than as told by a clinician or other third party. So we combed the research sent to us by the oncology content specialist, pulled out the patient quotes, reviewed open-ended interviews our company had previously conducted with cancer survivors, and conducted additional literature reviews, as needed, to have patient quotes from across the stages of the timeline.

I experienced overwhelming distress at my cancer’s recurrence with metastatic disease. I cried buckets of tears with my husband, family, and friends. However, the support of those who love me enough to supply companionship and food helped me realize that I wasn’t dying today. [18]

People say, ‘You have to be positive. You have to fight this.’ You’re sitting there, depressed, ill, and you just feel like saying, ‘I don’t feel positive.’ Then you feel guilty, ‘I should be positive to be healthy, but I don’t feel positive.’ There’s this whole Catch-22. [19]

We also identified a variety of sources, including national cancer websites such as the LiveStrong Foundation and the American Cancer Society, articles from magazines like “Coping with Cancer”, and newspapers, including “The New York Times”. Our criteria for choosing an article or patient story was simply that it had to contain actual patient quotes. We also limited our selection to quotes in which a cancer patient described his or her own experiences. In addition, we sought out patient quotes from research articles that included the recorded comments of cancer patients who otherwise might not have spoken out.

My spouse, and most of my family and friends are supportive, but they don’t seem to really understand the constant lifetime struggle of my cancer walk. … I feel my best when I’m around other cancer survivors. [20]

Initially . . . I couldn't put on earrings, hold a pencil, or button my pants with grace and dignity. Now, seven months out, I have full functionality but my fingertips feel waterlogged, like I've been swimming or hot-tubbing for too long. My doctor says that whatever you feel after a year will likely be permanent. [21]

We followed a process for affinity sorting, as outlined in *Mental Models: Aligning Design Strategy with Human Behavior* [22]. The affinity of the quotes was validated by a team of three user experience researchers. Strong parallels across cancer types were evident, including shock at hearing the diagnosis, difficulty choosing from conflicting treatment options, the waiting and uncertainty inherent in treatment, pressure to be positive, and the benefits of connecting with others who have experienced cancer. The map summarizes those common experiences.

Insights from our research led us to propose actionable strategies for writers and developers. With the assistance of the oncology content specialist, a family medicine physician, and a behavioral health psychologist, we identified the single most important behavior for each stage. For example, “Getting needed information and support” is the behavior for the treatment decisions stage. Because research supporting how best to move this behavior is cited, the map is not only a summary of user research but a resource for content or product development.

Translating research into user-centered mHealth apps is not an easy task. Multidisciplinary teams are composed of individuals with varying familiarity of the topic, varying understanding of user-centered design, and different geographical locations. Development proceeds on many parts separately. Tight timelines are the norm. The voice of the patient can easily lose prominence. We created the cancer experience map in part to bridge these gaps.

## The Cancer Experience Map

**Figure 1:**
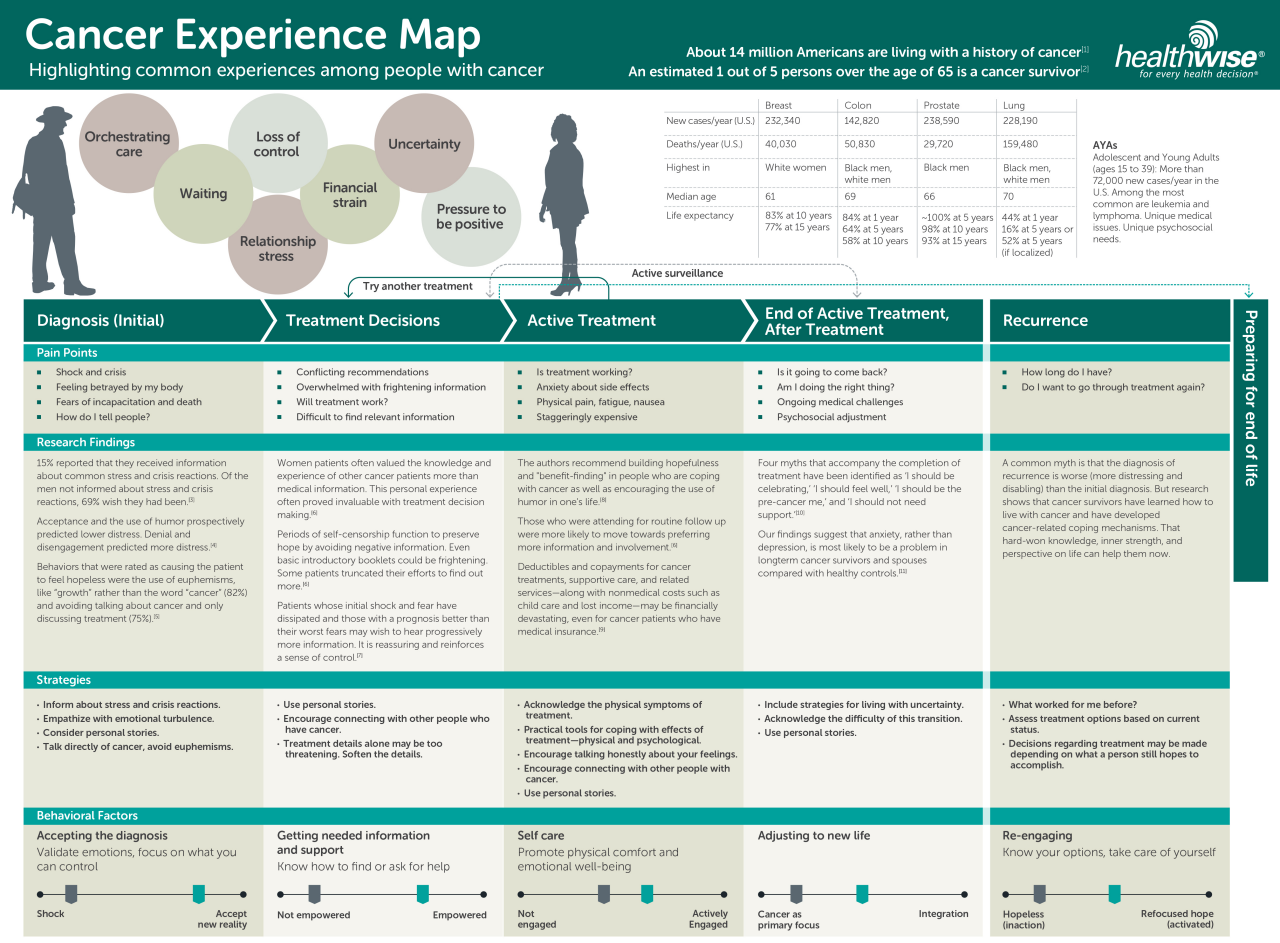
Cancer experience map (see Multimedia Appendix 1).

The cancer experience map represents the complexity of the cancer experience while capturing the common points of change and transition throughout. Based on direct quotes from cancer patients, the map provides design guidelines and identifies one behavioral factor for each stage. We found this combination to be an effective user-centered design strategy. The map is a tangible representation of the cancer patient voice, a solid and trustworthy resource for writers and developers tasked with creating supportive care materials for cancer patients. See Figure 1 and Multimedia Appendix 1.

### The Parts of the Map

The main part of the map (in shaded columns) is the experience timeline, which documents the continuum of stages from initial cancer diagnoses to possible recurrence.

Each stage is further broken down under the headings of Pain Points, Research Findings, Strategies, and Behavioral Factors.

- The Pain Points segment describes the very common situational stressors that someone in each stage is experiencing. For example, in the Diagnosis stage, people feel shock, fear of incapacitation and death, feel betrayed by their bodies, and wonder how to tell others about the diagnosis. In the Treatment Decisions stage, people grapple with conflicting treatment recommendations, get overwhelmed by frightening information, wonder whether a given treatment will work, and have a hard time finding relevant information to make an informed treatment choice. By referring to the pain points in a given stage, writers and developers can approach their work with a more holistic understanding of what the audience or end user is dealing with. Effective developers will keep in mind what is going on during the stage at which their product is aimed.
- The Research Findings segment lists cited information that supports our choice of pain points for each stage.
- The Strategies segment proposes best practices for addressing needs. For example, in the Diagnosis stage, shock and crisis are major stressors, yet research shows that only about 15% of testicular cancer patients report receiving information about common stress and crisis reactions [23]. This is clearly a gap, an unmet need for cancer patients. One of the strategies described, therefore, is to inform about common stress and crisis reactions and to empathize with emotional turbulence. Strategies are specific, actionable approaches to be considered by the developer of a solution for a given stage. The strategies vary significantly across the journey, just as the patient’s experience and needs evolve.
- The Behavioral Factors segment identifies the primary objective at each stage. These designations are based on expertise from our in-house behavioral health psychologists. For example, in the Diagnosis stage, the most important behavioral factor to influence is acceptance—moving from shock toward an acceptance of reality. Validating emotions and reminding someone to focus on what they *can* control are listed as ideas for moving a person in that direction.

Patterns that are not stage-specific and that show up throughout the journey include relationship stress, loss of control, and uncertainty. These journey-spanning patterns are represented by the colored circles at the top of the timeline.

The top right of the map includes a data table with cancer statistics, including a breakdown for the four most prevalent cancers: breast, colon, prostate, and lung. This macro research is included as a reference to complement the detailed experience timeline.

### How to Use the Map

A writer or developer creating materials or apps for cancer patients can refer to the map and see what a person is likely dealing with at each stage. Understanding stressors from the patient perspective, having research-based strategies, and knowing what behaviors to focus on: all of these help the writer or developer build effective products.

## The Medical Writer: Using the Map to Inform Cancer Support Videos

Knowing your audience is fundamental to writing good content. By listening to the words—the voices—of real people, developers can gain a better understanding of the audience they’re trying to reach. The cancer experience map—and the research that informed its development—helped give us insight into a large, diverse audience that would have otherwise been hard to represent.

The cancer experience map proved to be valuable for developing cancer support videos. The videos needed to be very short—at most three minutes—so we had to make hard decisions about what information to cut out or include to capture people’s experiences with cancer and to address cancer’s inherently difficult issues. The cancer experience map—and the verbatim quotes on which it is based—helped us decide what to focus on. The map helped to validate the choices we made about what would be most relevant to the most people.

Not everyone’s experience with cancer is the same. While there are commonalities, people tend to approach cancer in different ways, face different struggles, and find different ways of coping. Essentially, the map helped us answer the question: How can we make the videos more relatable—and helpful—to a greater number of people?

By reflecting both concerns and coping strategies relevant to each stage of the experience, the map helped us create more targeted, tailored videos that could speak to a person’s experience during different periods of time. The data also provided useful information on topics that aren’t as prevalent in evidence-based research, such as the role of spirituality.

After battling cancer, I have a real appreciation for trees, chipmunks, our dog, shrubs, flowers, clouds, people, thunderstorms, the stars at night and life itself—all these things seem more intense to me now. [24]

We also looked at various themes that emerged in the research, which were summarized in the map or, in many cases, represented by the actual patient quotes. They included the fear of the unknown, the pressure to act positive, the obligation to be strong for the sake of others, the importance of support from friends and family, ways of coping with difficult emotions, strength in one’s faith, and an appreciation for the small things in life. We were able to address many of these themes in the videos.

We approached these issues through personal stories. Through short vignettes, we were able to convey concepts—for example, focusing on what gives you strength—in a way that would resonate more with users. The details of the cancer experience map helped us make these stories richer and more nuanced and to capture the right emotional tone.

I am a firm believer in prayer. It calms me and gives me peace in times that I am spinning with emotions. It gives me someone to tell everything – however I want to say it – rather than picking the things that are appropriate for the person I’m talking to or working to say what I mean without seeming ungrateful or selfish or rude. [25]

The cancer experience map was a reliable guide for our approach to a difficult, complex subject. Our resulting set of 11 cancer support videos includes *Cancer: When You First Find Out, Cancer: Finding Your Strength*, and *Cancer: Life After Treatment.*

We want our cancer support videos to represent the patient voice as effectively as possible. And we want them to be shared with as many patients as possible. We are encouraged, knowing that policy can help make this happen.

## The Policy Advisor: Promoting the Patient Voice Through Health Care Policy

The imperative to include the patient voice in cancer supportive care content, whether through the use of the cancer experience map or other patient voice approaches, resonates as well upstream in the realm of health for all patients, that is, in health care policy.

Health care in the United States is in a time of great change and opportunity. At the heart of it, amidst all the swirl that accompanies a transition of this magnitude, is the patient. Many policy leaders, providers, payers, and other health care stakeholders who are active participants in this change agree that patient involvement is critical to the progress of health care. A noted thought leader blogged, “If patient engagement were a drug, it would be the blockbuster drug of the century and malpractice not to use it” [26].

While the goal is full participation by patients, where does it all begin? Perhaps the first step is with the relationship between a patient and his or her health care provider. Respect, mutual trust, and empathy all play a role in creating a relationship of professional intimacy, where shared decisions can be made.

The consequences for a patient can be significant when the provider and a patient do *not* share common understanding, goals, values, and assumptions—or said in another way, when the patient’s voice is not considered. Such a disconnect has negative consequences for both patient and provider, and ultimately, for the health care system at large, undermining whatever benefit, care, or healing is being sought or otherwise may be possible.

How can the importance of patient voice affect policy? The patient voice is a key to effective care, to systems that support that care, and to policy that enforces care. And the patient voice has already informed some policy in the United States. The Health and Human Services, Office of the National Coordinator (ONC) Health Information Standards Committee’s Consumer/Patient Engagement Power Team brought a group of patients and advocates together and asked them what they would want from Health Information Technology (HIT). This was the result—a goal to “ensure that pending regulatory requirements and standards meet current opportunities for engaging patients and their families in their care, and anticipate future policy and technology that encourages further engagement” [27].

This group made recommendations that will enable patients to participate as partners in their care. Their work has subsequently informed policy and standards work, specifically within the ONC for Health Information Technology Meaningful Use criteria. This includes patients’ access to medical records to view, download, or transmit (Blue Button); secure messaging; patient-generated data; patient-specific education in English and other languages; and further clarity on privacy and security for patients/consumers in the Health Insurance Portability and Accountability Act (HIPAA) omnibus rule.

Policy-focused groups (such as the ONC team) that represent the patient/consumer voice will continue to put pressure on access, design, education, quality, and care. This will affect innovative mHealth apps being created to provide cancer supportive care. Why is this important? The patient voice must be heard. The cancer experience map is an example of a guide, a design tool to help mHealth apps succeed.

## Discussion

We have described the development of the cancer experience map, which has value as a resource for mHealth app developers who are looking to include the voice and concerns of the cancer patient in the creation, development, and delivery of apps for cancer supportive care.

Cancer supportive care is an ideal focus for the development of mHealth apps. Cancer patients are a population of people who have been through a life-threatening and life-altering experience. In the United States, an estimated 14 million people live with a history of cancer. Many issues affect people who are in active treatment—hearing the diagnosis, making treatment decisions, dealing with side effects. Then another related but separate set of concerns arises after treatment: fear of recurrence, long-lasting or permanent effects from treatment, and the emotional and psychological consequences of going through such an experience. The transition in care from active treatment under an oncology care team back to a person’s former general practitioner can also be difficult for both patient and provider. Through all these stages, mHealth apps could offer seamless methods for patients to connect with their providers and vice versa. Issues such as how often checkups are needed or what symptoms are common or expected could be handled simply and quickly, and reassurance could replace worry or unneeded health care calls or office visits.

A major limitation to the current work is the lack of user-testing data thus far. User testing is important to find out if products are effective.

In our case, the cancer experience map was put to the test while the ink was still wet, so to speak. As the map was being assembled, our writer used parts of it in the scripts he wrote for the first six videos on coping with cancer. He then was able to use the entire cancer experience map for the next five scripts. However, the videos are still too recent to have accumulated enough user feedback to get a good sense of how they are perceived by viewers. Having sufficient user feedback will be key to knowing if we were successful in identifying commonalities in the cancer experience and addressing points of concern. We look forward to gathering this additional data.

Another limitation we encountered is that projects like the cancer experience map are ordinarily featured in product development materials rather than professional research journals; consequently, this article lacks a systematic literature review.

While the cancer experience map reflects the experience of Americans with cancer, it is likely that some of these traits are universal. Further research is needed to discover what those similarities and differences are and to see how our work compares with similar research projects in other parts of the world.

## Conclusion

The cancer experience map presented in this paper is one way mHealth app developers can consider and include the voice of the cancer patient in their design, creation, testing, and utilization of apps for cancer supportive care. This is an exciting time for mHealth app developers, as rapid developments in technology are moving beyond those that seemed so novel just yesterday, and apps that offered limited fitness data or diet information are giving way to wearable motion sensor detectors and programs that seamlessly integrate mHealth apps with electronic health records systems. It is only a matter of time before current obstacles, such as security concerns and certification criteria, are resolved. With this article, we offer a contribution—the cancer experience map—to help developers create cancer supportive care apps that will assure patients that their voice is being heard.

## Multimedia Appendix 1

Cancer experience map.

## Abbreviations

ONC: Office of the National Coordinator, Health and Human Services
