# Supplementary material for: The Cancer Experience Map: An Approach to Including the Patient Voice in Supportive Care Solutions
Source: J Med Internet Res. 2015 May 28;17(5):e132. doi: 10.2196/jmir.3652 (PMC4468569; doi:10.2196/jmir.3652)
Supplement: Supplementary file 1 [file jmir_v17i5e132_app1.pdf]

Cancer Experience Map

Highlighting common experiences among people with cancer

About 14 million Americans are living with a history of cancer[1]
An estimated 1 out of 5 persons over the age of 65 is a cancer survivor[2]

healthwise® for every health decision®

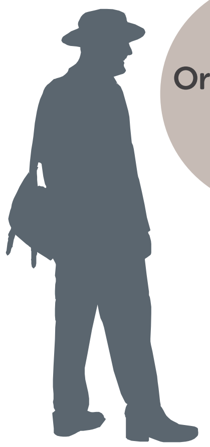

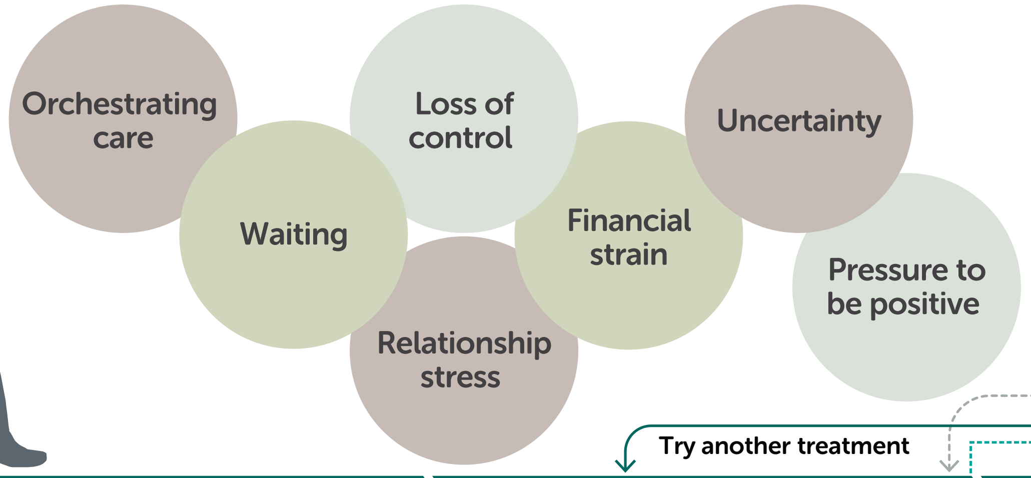

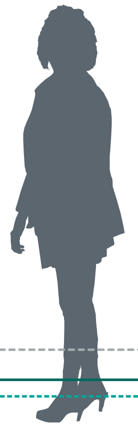

	Breast	Colon	Prostate	Lung
New cases/year (U.S.)	232,340	142,820	238,590	228,190
Deaths/year (U.S.)	40,030	50,830	29,720	159,480
Highest in	White women	Black men, white men	Black men	Black men, white men
Median age	61	69	66	70
Life expectancy	83% at 10 years<br>77% at 15 years	84% at 1 year<br>64% at 5 years<br>58% at 10 years	~100% at 5 years<br>98% at 10 years<br>93% at 15 years	44% at 1 year<br>16% at 5 years or 52% at 5 years (if localized)

AYAs
Adolescent and Young Adults (ages 15 to 39): More than 72,000 new cases/year in the U.S. Among the most common are leukemia and lymphoma. Unique medical issues. Unique psychosocial needs.

Try another treatment

Active surveillance

Diagnosis (Initial) → Treatment Decisions → Active Treatment → End of Active Treatment, After Treatment → Recurrence → Preparing for end of life

Pain Points

Diagnosis (Initial)

- Shock and crisis
- Feeling betrayed by my body
- Fears of incapacitation and death
- How do I tell people?

Treatment Decisions

- Conflicting recommendations
- Overwhelmed with frightening information
- Will treatment work?
- Difficult to find relevant information

Active Treatment

- Is treatment working?
- Anxiety about side effects
- Physical pain, fatigue, nausea
- Staggeringly expensive

End of Active Treatment, After Treatment

- Is it going to come back?
- Am I doing the right thing?
- Ongoing medical challenges
- Psychosocial adjustment

Recurrence

- How long do I have?
- Do I want to go through treatment again?

Research Findings

Diagnosis (Initial)

15% reported that they received information about common stress and crisis reactions. Of the men not informed about stress and crisis reactions, 69% wish they had been.[3]

Acceptance and the use of humor prospectively predicted lower distress. Denial and disengagement predicted more distress.[4]

Behaviors that were rated as causing the patient to feel hopeless were the use of euphemisms, like "growth" rather than the word "cancer" (82%) and avoiding talking about cancer and only discussing treatment (75%).[5]

Treatment Decisions

Women patients often valued the knowledge and experience of other cancer patients more than medical information. This personal experience often proved invaluable with treatment decision making.[6]

Periods of self-censorship function to preserve hope by avoiding negative information. Even basic introductory booklets could be frightening. Some patients truncated their efforts to find out more.[6]

Patients whose initial shock and fear have dissipated and those with a prognosis better than their worst fears may wish to hear progressively more information. It is reassuring and reinforces a sense of control.[7]

Active Treatment

The authors recommend building hopefulness and "benefit-finding" in people who are coping with cancer as well as encouraging the use of humor in one's life.[8]

Those who were attending for routine follow up were more likely to move towards preferring more information and involvement.[6]

Deductibles and copayments for cancer treatments, supportive care, and related services—along with nonmedical costs such as child care and lost income—may be financially devastating, even for cancer patients who have medical insurance.[9]

End of Active Treatment, After Treatment

Four myths that accompany the completion of treatment have been identified as 'I should be celebrating,' 'I should feel well,' 'I should be the pre-cancer me,' and 'I should not need support.'[10]

Our findings suggest that anxiety, rather than depression, is most likely to be a problem in longterm cancer survivors and spouses compared with healthy controls.[11]

Recurrence

A common myth is that the diagnosis of recurrence is worse (more distressing and disabling) than the initial diagnosis. But research shows that cancer survivors have learned how to live with cancer and have developed cancer-related coping mechanisms. That hard-won knowledge, inner strength, and perspective on life can help them now.

Strategies

Diagnosis (Initial)

- **Inform about stress and crisis reactions.**
- **Empathize with emotional turbulence.**
- **Consider personal stories.**
- **Talk directly of cancer, avoid euphemisms.**

Treatment Decisions

- **Use personal stories.**
- **Encourage connecting with other people who have cancer.**
- **Treatment details alone may be too threatening. Soften the details.**

Active Treatment

- **Acknowledge the physical symptoms of treatment.**
- **Practical tools for coping with effects of treatment—physical and psychological.**
- **Encourage talking honestly about your feelings.**
- **Encourage connecting with other people with cancer.**
- **Use personal stories.**

End of Active Treatment, After Treatment

- **Include strategies for living with uncertainty.**
- **Acknowledge the difficulty of this transition.**
- **Use personal stories.**

Recurrence

- **What worked for me before?**
- **Assess treatment options based on current status.**
- **Decisions regarding treatment may be made depending on what a person still hopes to accomplish.**

Behavioral Factors

Accepting the diagnosis
Validate emotions, focus on what you can control
Shock — Accept new reality

Getting needed information and support
Know how to find or ask for help
Not empowered — Empowered

Self care
Promote physical comfort and emotional well-being
Not engaged — Actively Engaged

Adjusting to new life
Cancer as primary focus — Integration

Re-engaging
Know your options, take care of yourself
Hopeless (inaction) — Refocused hope (activated)

Licensed under the Creative Commons Attribution License - Any reproductions of the Cancer Experience Map requires inclusion of this note and the full citation of the source. Please cite as:

Hall LK, Kunz BF, Davis EV, Dawson RI, Powers RS
The Cancer Experience Map: An Approach to Including the Patient Voice in Supportive Care Solutions
J Med Internet Res 2015;17(5):e132
URL: http://www.jmir.org/2015/5/e132/
doi:10.2196/jmir.3652

Printed wallcharts and postcards with the map can be ordered at JMIR Publications, please contact jmir.editorial.office@gmail.com for a quote.

References cited in the map:

1. American Cancer Society. Cancer Facts & Figures 2014. Atlanta: American Cancer Society; 2014

2. National Comprehensive Cancer Network (2014). Survivorship. NCCN Clinical Practice Guidelines in Oncology, version 2.2014. http://www.nccn.org/professionals/physician_gls/pdf/survivorship.pdf

3. Skoogh J, Steineck G, Johansson B, Wilderäng U, Stierner U, & SWENOTECA. Psychological needs when diagnosed with testicular cancer: findings from a population-based study with long-term follow-up. BJU Int. 2013 Jun;111(8):1287-93. PMID: 23469865

4. Carver CS, Pozo C, Harris SD, Noriega V, Scheier MF, Robinson DS, Ketcham AS, Moffat FL Jr, Clark KC. How coping mediates the effect of optimism on distress: a study of women with early stage breast cancer. J Pers Soc Psychol. 1993 Aug;65(2):375-90. PMID: 8366426

5. Hagerty RG, Butow PN, Ellis PM, Lobb EA, Pendlebury SC, Leighl N, MacLeod C, Tattersall MH. Communicating with realism and hope: incurable cancer patients' views on the disclosure of prognosis. J Clin Oncol. 2005 Feb 20;23(6):1278-88. PMID: 15718326

6. Leydon GM, Boulton M, Moynihan C, Jones A, Mossman J, Boudioni M, McPherson K. Faith, hope, and charity: an in-depth interview study of cancer patients' information needs and information-seeking behavior. West J Med. 2000 Jul;173(1):26-31. PMID: 10903285

7. Butow PN, Maclean M, Dunn SM, Tattersall MH, Boyer MJ. The dynamics of change: cancer patients' preferences for information, involvement and support. Ann Oncol. 1997 Sep;8(9):857-63. PMID: 9358935

8. Shapiro JP, McCue K, Heyman EN, Dey T, Haller HS. Coping-Related Variables Associated with Individual Differences in Adjustment to Cancer. J Psychosoc Oncol. 2010;28(1):1-22. PMID: 20391063

9. Rucke K. 2013. Americans Diagnosed With Cancer More Than Twice As Likely To Go Bankrupt. MintPress News. http://www.mintpressnews.com/americans-diagnosed-with-cancer-more-than-twice-as-likely-to- declare-bankruptcy/160227/. Archived at: http://www.webcitation.org/6QbJXStLI

10. Waldrop DP, O'Connor TL, Trabold N. "Waiting for the other shoe to drop:" distress and coping during and after treatment for breast cancer. J Psychosoc Oncol. 2011;29(4):450-73. PMID: 21966727

11. Mitchell AJ, Ferguson DW, Gill J, Paul J, Symonds P. Depression and anxiety in long-term cancer survivors compared with spouses and healthy controls: a systematic review
